# Screening of Foreign Direct Investment and the States’ Security Interests in Light of the OECD, UNCTAD and Other International Guidelines

**DOI:** 10.1007/978-3-030-58916-5_10

**Published:** 2021-03-04

**Authors:** Pascale Accaoui Lorfing

**Affiliations:** French National Centre for Scientific Research (CNRS)-CERSA, University Paris II Panthéon-Assas, Paris, France; CREDIMI, University of Burgundy, Dijon, France

**Keywords:** National security interest, Security measures, Public order, Foreign investment, Bilateral investment treaties (BITs), International investment agreements (IIAs), OECD-UNCTAD Guidelines, Santiago Principles on Sovereign Wealth Funds (SWFs), Customary international law

## Abstract

This chapter analyses the concept of the “national security interest”, which is widely recognised as allowing a state to determine which areas of its economy are restricted or prohibited to foreign investors. This chapter seeks to identify what constitutes a threat for a state and how that threat is managed both domestically and internationally. Despite the recognition of a state’s right to take measures it considers essential to its security, there are limits. The rules established by the Organisation for Economic Co-operation and Development (OECD) and the United Nations Conference on Trade and Development (UNCTAD) and other international instruments are non-binding but can serve as a guide for states in determining the limits of the national security approach. International investment agreements can restrict the right of states to take security-related measures. Finally, customary international law, in light of the good faith obligation, can serve as a basis for assessing measures taken by a state and pave the way for a better balance between the rights of a state and those of foreign investors.

## Introduction

States seek to protect their economy from foreign investment if the latter poses a threat to their national security. This mechanism takes the form of state measures regarding the access of foreign investment to certain categories of the national economy. Although not new, this mechanism seems to be gaining momentum since 2010. A recent report by the Organisation for Economic Co-operation and Development (OECD) and the United Nations Conference on Trade and Development (UNCTAD) has documented the increase in national security measures and has predicted that the trend will continue.^1^ Indeed, the liberalisation of the world economy in the 1980s led to an increase in foreign investment. As a result, foreign investment has flown in areas of vital interest to host states, particularly in areas such as energy and infrastructure. This has made recipient countries aware of the fact that such foreign investments can potentially endanger entire economic areas and jeopardise their independence. These foreign investments are therefore likely to be subject to control by the host country.

The term “foreign investment” may refer to private foreign investment, sovereign wealth funds (SWFs) or state-owned enterprises (SOEs),^2^ the latter being new categories of institutional investors who are engaged in commercial activities. The difference between private foreign investors and SWFs or SOEs is that the latter are created, controlled and financed by the home state.^3^

National screening measures can have an impact on foreign investment as they can restrict or prohibit foreign investors from investing in strategic sectors. International investment agreements (IIAs) in general, including some of the most recent IIAs,^4^ recognise national security concerns and the right of a state to take measures for the protection of its essential security interests. The issue then becomes one of determining what constitutes a legitimate national security interest and its limits. The chapter will consider these two issues in turn.

## National Security Interest

In order to address legitimate national security interests, a state will identify the risks incurred in relation to the foreign investor (Sect. 2.1) and specify how it intends to manage that risk (Sect. 2.2).

### Risk Identification

States identify risks as a threat to their integrity, whether economic or political. Risks may evolve as a host state’s environment changes, i.e., as its geopolitical environment and security situation evolve. In the past, threats were related to espionage. Today, threats concern wider areas. They may be related to the military or natural resources. They may also come from control over the acquisition or ownership of certain sensitive assets, or from investments in critical infrastructure, such as electricity and distribution, railways and water supply.^5^ Risk can generally be limited to certain vital sectors of a host state or it may relate to natural resources, such as energy. It may also concern transport, fisheries, broadcasting, technology and telecommunications, or other advanced technologies. The last area of risk is related to personal data and companies that control the data.^6^

The identification of a risk may vary from state to state. In Lithuania, for example, sectors considered key to national security are those related to energy, transport, information, technology and telecommunications, finance and credit, and military equipment.^7^ In Australia, the sensitive sectors include mining and agricultural land.^8^

Specific risks, however, are associated with SWFs^9^ and SOEs^10^ and fall into two categories: political risks and economic risks.^11^ The political risks stem from the fact that SWFs and SOEs may serve the policy of their home state, and not simply pursue a purely economic objective, leading to the adoption by the host state of specific requirements related to activities of such investors.^12^ The economic risks are likely to result from the potential impact of SWFs and SOEs on the host state’s, market, which may create financial instability. Foreign investors may become a significant shareholder or owner of a sector of a state’s economy. Examples include the purchase of the port of Piraeus by the Chinese company COSCO under the Chinese Belt & Road Initiative (BRI)^13^ or, even though less fortunate, if one may say so, the failed attempt by the China National Offshore Oil Corporation to acquire Unocal, a US oil company.^14^

### Risk Management

Risks resulting from foreign investment in certain sectors of a host state’s economy are therefore managed by the host state. Policies adopted by the host state to protect national interests may be taken at the national level (Sect. 2.2.1) or at the international level (Sect. 2.2.2).

#### Risk Management at the National Level

A recent OECD-UNCTAD study shows that state policies to “safeguard essential security include a broadening of the scope of transactions that are subject to review—in particular to include assets that provide the acquirer access to sensitive personal data and advanced technology—, an extension of the timeframe for the screening process and a lowering of trigger thresholds to also include smaller investments and stakes”.^15^ In a first step, this chapter will examine some general screening measures. In a second step, it will consider the recent impact of COVID-19 on screening measures.

##### General Screening Measures

A few examples illustrate risk management at the national level.^16^ France is one of the states that have already modified their policies. In 2012, France drew a list of sectors requiring prior authorisation for foreign investors.^17^ It modified its foreign investment screening mechanism in order to safeguard its essential security interests. In recent legislation, which entered into force on 1 January 2019,^18^ France notably restricted access to foreign investors by identifying sectors that require authorisation for foreign investors and reserving the right to refuse a foreign investment.^19^ For its part, the revised German Foreign Trade and Payments Ordinance, which entered into force on 29 December 2018, established a revised screening procedure requiring a lower voting threshold for acquisitions by non-European investors and changes to the review procedures in sensitive sectors, such as key infrastructure and defence-related industries.^20^ In 2012, Italy set up a review mechanism for transactions in specific sectors (defence or national security, strategic activities in the field of energy, transport and communications)^21^ and, in 2019, it extended the list of strategic assets requiring notification to the government of any contract related to the list.^22^ Under Federal Law No. 155-FZ of 1 July 2017,^23^ the Russian Federation excluded certain foreign investors from participating in the privatisation of state and municipal property, while Canada’s Guidelines on the National Security Review of Investments (2016) are related to national security and intend to provide greater clarity to existing policies.^24^

In 2008, Australia revised its 1975 Foreign Acquisitions and Takeover Act^25^ to add foreign investment control tools and criteria. In 2010, Australia introduced restrictions on investment in the mining sector.^26^ In February 2018, it clarified the review process for acquisitions in certain sectors, such as distribution and electricity transmission, and stated, for security reasons, that conditions and restrictions on foreign acquisitions and takeovers may be required on a case-by-case basis.^27^ South Africa’s Competition Amendment Act 2018 authorises the South African President to “constitute a national security review committee on foreign investments which will conduct mandatory reviews of inward foreign investment to safeguard South Africa’s essential security interests. Sectors as well as critical infrastructure assets to which the review mechanism will apply are to be listed”.^28^

In the European Union, the Regulation of the European Parliament and of the Council establishing a framework for the screening of foreign direct investment in the Union entered into force on 10 April 2019 and has been fully operational since 11 October 2020.^29^ The regulation sets in place a mechanism for the exchange of information between member states enabling them to review a foreign investment for reasons of public order and security. The Regulation also allows the European Commission to give non-binding opinions where a foreign investment may pose a potential threat to essential security interests of more than one member state or the European Union as a whole.^30^

In August 2018, the US Congress passed the Foreign Investment Risk Review Modernization Act (FIRRMA), which expands the jurisdiction of the Committee on Foreign Investment in the United States (CFIUS) to review investments or acquisitions by foreign investors in US companies. It does so by including in its jurisdiction new categories of transactions involving foreign investors when matters of national interest are at stake, such as real estate transactions, changes in foreign investor rights and potential foreign control of a US business.^31^

CFIUS’ filings related to “critical technologies and in certain industries” are also made mandatory, alongside the voluntary filing system for transactions involving foreign investors.^32^ The terms are defined broadly to take account of any situation involving a foreign investment that may be detrimental to the national interest. The Final Regulations implementing FIRRMA came into force on 13 January 2020, as the Treasury Department issued two final regulations implementing changes to CFIUS’ jurisdiction and process.^33^ The first regulation consists of provisions pertaining to certain investments in the US by foreign persons.^34^ The second regulation comprises provisions pertaining to certain transactions by foreign persons involving real estate in the United States.^35^

Moreover, as SOEs and/or SWFs can endanger strategic industries, states have introduced additional screening requirements.^36^ In Australia, for example, SOEs are subject to extensive disclosure requirements and their investments subject to prior governmental consent.^37^ In the Russian Federation, SOEs are subject to prior approval for transactions involving minority shareholdings in domestic enterprises and are prohibited from acquiring majority shareholdings.^38^ Similarly, the latest revision of the French legislation on foreign investment requires investors to disclose their links with a foreign state or public body.^39^

##### Recent Impact of COVID-19 on Screening Measures

The COVID-19 outbreak in early 2020 created a public health emergency with social and economic repercussions that caused a major economic shock. The pandemic introduced a potential risk to strategic industries which has led to new considerations related to the protection of citizens at EU level and at national level.^40^

The European Commission recalled the importance of protecting EU citizens, industry and economy by preserving production and value chains, by ensuring the necessary supplies to health systems, jobs and liquidity, and by protecting EU citizens from “predatory buying” of strategic assets by foreign investors.^41^ In this context, the European Commission invited EU member states to adopt measures to protect their citizens and industries by providing guidance concerning foreign direct investment flows.^42^ Accordingly, the Commission stated that EU member states “*need to be vigilant and use all tools available at Union and national level to avoid that the current crisis leads to a loss of critical assets and technology.* This includes tools like national security screening and other security related instruments. The Commission will guide Member States ahead of the application of the Foreign Direct Investment Screening Regulation”.^43^

At the national level, the Commission encouraged EU member states to use their existing FDI screening mechanisms to counter potential risks related to health infrastructure, supply, and “other critical sectors as envisaged in the EU legal framework”.^44^ EU member states should also adopt measures to restrict capital movements as needed to protect strategic assets from foreign investors’ “predatory buying” pursuant to Article 65(1)(b) of the Treaty on the Functioning of the European Union (TFEU) that establishes the right of member states “to take measures which are justified on grounds of public policy or public security”.^45^ For state members that do not yet have a screening mechanism^46^ and those with a screening mechanism that does not take into account these specific risks, the Commission argues in favour of the creation of “a full-fledged screening mechanism and in the meantime to use all other available options to address cases”.^47^ The screening mechanism may result either in the prohibition of foreign investment in certain industries or in limiting it, such as by imposing compulsory licences.^48^

India too has introduced restrictions on FDI policy to protect the country from takeovers and/or acquisitions of Indian companies during the COVID-19 pandemic. In this regard, the Government has issued a Press Note setting out restrictions and requiring prior government approval for any investment to be made within Indian territory and prohibiting investment in certain areas (defence, space, atomic energy, …).^49^

The link between COVID-19 and the change in approach to FDI is obvious. The new policy aims to protect the state from FDI that may endanger domestic industries, which the state considers to be in its national interest to protect. The tool to be deployed is the screening mechanism. This approach is based on the need to take into account considerations other than economic interests, such as the protection of public health, states’ awareness of the threat and the urgency of the measures to be taken in the national interest.

#### Risk Management at the International Level

The management of the risk of a threat to national security interests is also present at the international level, where two aspects can be taken into account. The first aspect concerns the diverse formulations of what constitutes a threat to a host state as incorporated in IIAs. The second aspect is the variety of approaches adopted by IIAs to address the issue.

##### Various Formulations of “Threat” in IIAs

IIAs refer to a threat to the national security interests of a host state in several ways.^50^ Some IIAs, such as the bilateral investment treaty (BIT) between Hungary and the Russian Federation (1995) use the term “national security”.^51^ Other IIAs, such as the Indian-Singapore Economic Cooperation Agreement (2005) use instead the term “essential security interests”.^52^ Alternatively, other IIAs use the term “public order”^53^ which can also be combined with the term “essential security interests” to point to a threat to national security. This is the choice made by the Recommendation of the OECD Council on “Member country measures concerning National Treatment of foreign-controlled enterprises in OECD member countries and based on considerations of public order and essential security interests”.^54^ The German Model BIT (2009) also uses this wording. It provides that “[m]easures that have to be taken for reasons of public security and order, shall not be deemed treatment less favourable within the meaning of this Article”.^55^ This is also the approach adopted in the Estonia-United States BIT (1994) which states: “This Treaty shall not preclude the application by either Party of measures necessary for the maintenance of public order, the fulfilment of its obligations with respect to the maintenance or restoration of international peace or security, or the protection of its own essential security interests”.^56^

##### Variety of IIA Approaches to the Protection of National Interests

In contrast with older generation BITs that do not generally include essential security interests exceptions, recent IIAs tend to do so.^57^ The following paragraphs will focus on recent IIAs that provide for the protection of the states’ national interests. Two approaches seem to emerge: a broad and a narrow approach.

Host states may adopt a broad approach in the drafting of the provision on national security inserted in an IIA, allowing for a wide range of measures to be covered. For instance, the exception may allow the host state to take measures “for its national interests” without qualifying the policy fields concerned. An example is given by Article 18 on “Essential Security” of the US Model BIT (2012) which states that:

Nothing in this Treaty shall be construed:

1. to require a Party to furnish or allow access to any information the disclosure of which it determines to be contrary to its essential security interests; or
2. to preclude a Party from applying measures that it considers necessary for the fulfilment of its obligations with respect to the maintenance or restoration of international peace or security, or the protection of its own essential security interests.

A broad approach may also provide for the right of a state to take measures for the protection of its national interest in specific fields but in general terms. Article 2(3) of the BIT between Russia and Hungary, which entered into force in 1996, recognises the right of a state to take measures “necessary for the maintenance of defence, national security and public order, protection of the environment, morality and public health”.

Finally, the broad approach is even more evident in cases where security measures are excluded from the scope of the dispute settlement provision. Article 19 of the Austria-Mexico BIT on “Exclusions”, gives the following example:

The dispute settlement provisions of this Part shall not apply to the resolutions adopted by a Contracting party which, for national security reasons, prohibit or restrict the acquisition of an investment in its territory, owned or controlled by its nationals, by investors of the other Contracting party, according to the legislation of each Contracting Party.

This provision establishes in very clear terms that measures adopted by the state in its national interest *and* related to the specific policy decisions are excluded from the scope of the dispute settlement clause. The provision deprives therefore the tribunal of jurisdiction in the circumstances listed. The extraordinary measure taken on grounds of national security which excludes the jurisdiction of the tribunal should be expressly worded to be taken into account.^58^

When the essential security interests provision establishes that the state may take the measures “that it considers necessary” the broad approach gives the state a discretion to determine what is related to its national interest and the exception is deemed to be of self-judging character.^59^ The broad wording of a self-judging “national interest” clause raises the question of whether the state has complete discretion in interpreting it. This question has been the subject of debate in the case law and in legal scholarship.^60^ Normally, the national security measure will be open to scrutiny by the tribunal according to the test of proportionality, i.e. the reasonableness of the measure in its context,^61^ and it will remain subject to a limited good faith review.^62^

Alternatively, host states may take a narrow approach to formulating IIA provisions on the protection of their national security interests. The narrow approach covers exceptions for measures a state takes to protect its “essential security interests” in one or more specific policy fields or sectors. The narrow approach may also define the precise conditions under which the “vital interest” of a state may be invoked, whether, for example, in the military sector or in a specific industry.

An example is provided by Article 18(4)(b) on “General Exceptions” of the Canadian Model BIT (2014) which reads as follows:

This Agreement does not

- prevent a Party from taking an action that it considers necessary to protect its essential security interests:
  (i) relating to the traffic in arms, ammunition and implements of war and to such traffic and transactions in other goods, materials, services and technology undertaken directly or indirectly for the purpose of supplying a military or other security establishment,
  (ii) taken in time of war or other emergency in international relations, or
  (iii) relating to the implementation of national policies or international agreements respecting the non-proliferation of nuclear weapons or other nuclear explosive devices; […].

The Canadian provision is limited in scope as it identifies areas in relation to which the state may take measures to protect its essential security interests (arms trafficking, during wartime, or nuclear non-proliferation). This provision is of a self-judging character as the state will determine what “it considers necessary” in the enumerated areas. The same approach is taken in Article 2102 of the North American Free Trade Agreement (NAFTA) on “National Security”^63^ in Chapter XXI on “Exceptions”. Other instruments adopt the same approach such as the General Agreement on Trade in Services (GATS) in Article XIV (bis).^64^

## Limits to the National Security Interest

Irrespective of the approach chosen, the right of a state to take security-related measures is subject to certain limits. Limits on a state’s power to invoke the national security interest may include those set by the OECD and other guidelines and principles (Sect. 3.1), IIAs (Sect. 3.2) and customary international law (Sect. 3.3).

### The OECD and Other Guidelines and Principles

The OECD has issued a number of instruments with the aim of recommending tools that states can use when they adopt concrete measures based on considerations of public order and essential security interests. The OECD Codes of Liberalisation of Capital Movements (2019) and Liberalisation of Current Invisible Operations^65^ expressly recognise the right of each OECD member state to take measures which it considers necessary for the protection of its essential security interests.^66^

Adopted on 21 June 1976, revised periodically and last updated in 2011, the OECD Declaration on International Investment and Multinational Enterprises recalls the importance of multinational enterprises in the field of international investment. The OECD Declaration stresses the need for international cooperation between a host state and multinational enterprises. The principles of transparency, national treatment and consultation must be respected in order to minimise or avoid occasions when host states impose conflicting requirements on multinational enterprises.

Adopted on 16 July 1986, the OECD Recommendation on Member Country Measures concerning National Treatment of Foreign-Controlled Enterprises in OECD Member Countries and based on Consideration of Public Order and Essential Security Interests, recommends transparency of such measures when they are notified to the OECD.^67^ This OECD recommendation invites adhering countries to limit the use of national treatment measures for foreign-controlled enterprises to areas where public policy and essential security interests are at stake. The Recommendation also invites adhering countries to narrow the scope of these measures by adopting alternative regulations that would allow foreign-controlled enterprises to operate in the host state.

The OECD Recommendation on the National Treatment Instrument also encourages host states to ensure that foreign-controlled enterprises operating in their territory are treated no less favourably than domestic enterprises.^68^ Where a host state considers that the foreign investment constitutes a threat to its national security interests, the Recommendation establishes the procedure consisting of (1) a declaration of principle by adhering countries, (2) notification of their exception to the OECD and (3) a monitoring procedure to deal with such an exception within the OECD. Although the National Treatment Instrument is a non-binding voluntary commitment by both adhering and non-adhering countries to the OECD, its purpose is to treat measures taken by a host state in its national interest as an exception. This exception is limited in nature and scope.

In the same vein, the OECD Guidelines for Recipient Country Investment Policies relating to National Security (2009) establish non-binding principles.^69^ Their purpose is to set out the exceptional nature of the measure taken in the national interest. The Guidelines also aim to avoid protectionism that may result from the introduction by a state of national policies aimed to safeguard national security interests. The Guidelines, such as the principles of non-discrimination, transparency and predictability, proportionality of measures and accountability of implementing authorities, are intended to guide states in adopting measures in the national interest.^70^ The requirement of non-discrimination means that the measures taken must be of “general application” treating “similarly situated investors in a similar fashion” and they must be “taken with respect to individual investments based on specific circumstances of the individual investment which pose a risk to a national security”.^71^ The requirements of transparency and predictability imply that the measures taken must be made public by all means, whether in a public register^72^ or on the internet, with an assessment of the criteria made available to the public. This includes prior notification to the interested party of proposed changes to investment policies, consultation with the other parties, and, at the procedural level, the establishment of strict time limits for the review of foreign investment screening procedures and the protection of commercially sensitive information of foreign investors. Furthermore, disclosure of investment policy measures should be made through press releases, annual reports and/or reports to parliament, bearing in mind the obligation to protect sensitive and classified information.^73^

The requirement of regulatory proportionality means that national security measures must link investment restrictions and risks to national security. It also means that particular attention must be paid to how the national interest exception is drafted and interpreted. As noted above, the broad approach to the provision on security interests gives the state greater flexibility in determining what is necessary to protect its national security. The proportionality requirement is therefore useful as it sets limits and provides guidance that a state should take into account.^74^ Finally, a state is accountable to its citizens. The state is also required to adopt international accountability mechanisms and grant foreign investors the possibility of a recourse against it.^75^

In the same vein, the G20 Guiding Principles for Global Investment Policymaking (2016) establish non-binding principles of transparency and coherence, among others, to guide investment policymaking. These instruments recognise the importance for a state to take measures for its national security. As recommendations, they can serve as a guide to states when taking measures to safeguard national security interests in order to limit their impact on investment flows.

Finally, with regard to SWFs, the Santiago Guidelines, also known as Generally Accepted Principles and Practices (GAPP), adopted on 11 October 2008, are a voluntary set of 24 best practice guidelines for SWF operations.^76^ Their objective is to maintain (1) a stable global financial system, (2) adequate risk control and (3) a regulatory structure. They are seen as common international standards of transparency, independence and governance that all SWFs can follow.

### Limits Set Out in IIAs

Limits to the state’s national security interests also derive from the limits set out in international investment agreements. The right of a state to adopt security-related measures is likely to be limited by IIAs. This is particularly true in the absence of a provision on “security interests” in the IIA (Sect. 3.2.1), where the security related provision is narrowly drafted (Sect. 3.2.2) or where state parties exclude certain areas from the provision on “security interests” (Sect. 3.2.3).

#### The Absence of a Provision on “Security Interests” in IIAs

According to a 2009 UNCTAD study, only a few BITs include a national security interests’ exception.^77^ The absence of any provision on “security interests” in BITs limits the right of a state to take security-related measures. Accordingly, the impact of the state measure taken for reasons of public interest will be assessed by an arbitral tribunal in the light of the investor protection provisions of the applicable BIT. In other words, the absence of a provision on “security interests” in BITs or free trade agreements (FTAs) with an investment chapter allows the investor to be better protected against state action related to essential security interests.

#### The Restrictive Wording of the Provision on “Security Interests”

A state’s right to adopt security measures is also limited when the security-related exception is drafted in a restrictive manner, such as in the case of the previously-discussed Canadian Model BIT, which limits what may constitute a security interest to trafficking in arms, munitions and war material, and the non-proliferation of nuclear weapons.^78^ If a related claim is brought before an arbitral tribunal, the latter will then assess the state measure in light of the wording of the provision of the BIT. Any security-related state measure that is outside the scope of the provision on “essential security interests” will be assessed in light of the standards of protection in IIAs and is likely to lead to state responsibility.

#### The Limited Application of “Essential Security Interests” to Specific Provisions

A state’s right to take security-related measures is ultimately limited by the scope of the treaty’s exceptions. For instance, this will be the case when only “expropriation or nationalization or similar measures” are covered by the scope of the treaty’s exceptions, provided that other conditions specified in the clause are met.^79^ For example, the China-Philippines BIT states:

1. Either Contracting Party may for reasons of national security and public interest, expropriate, nationalize or take similar measures (hereinafter referred to as “expropriation”) against investments of investors of the other Contracting party, but the following conditions shall be met:

1. under domestic procedure;
2. without discrimination
3. upon payment of fair and reasonable compensation.^80^

Where the state undertakes, in a BIT or FTA, to limit its right to adopt security-related measures to a specific provision, such as that relating to expropriation, it can no longer adopt measures outside the scope of the specific clause without incurring liability.

### Customary International Law

Limitations on a state’s adoption of security-related measures also arise under customary international law. Customary international law may play an important role in assessing a state’s security-related measures, even in the presence of an essential security interests exception. We will consider the example of the World Trade Organization (WTO) panel report in *Russia – Measures concerning traffic in transit* of 5 April 2019 in the context of a dispute between Russia and Ukraine.^81^ The questions dealt with allow for an analogy with the subject of our chapter. The WTO Panel addressed the interpretation of Article XXI(b)(iii) of the 1994 General Agreement on Tariffs and Trade (GATT) on “Security Exceptions”,^82^ the measures at issue and their existence,^83^ whether the measures were “taken in time of war or other emergency in international relations”, and whether the conditions of the introductory part^84^ of Article XXI(b) of the GATT were satisfied.^85^

With regard to the facts and very briefly, in its dispute with Ukraine, the Russian Federation invoked the national security exception in Article XXI of the GATT concerning restrictions on road and rail transit traffic through the territory of the Russian Federation.^86^ The Russian Federation justified the national security exception as being necessary for the protection of its essential security interests. The Panel analysed Article XXI of the GATT in light of the principle of good faith as codified in the Vienna Convention on the Law of Treaties.^87^ In the Panel’s view, Article XXI of the GATT provides for “essential security interests” in a strict sense, understood as being limited to the “quintessential functions of the state”.^88^ The Panel further recognises the right of “every member to define what it considers to be its essential security interests”.^89^ However, the state is not free to raise any concerns as “essential security interests”. This right is limited by the principle of good faith^90^ which applies, on the one hand, to the interpretation of what constitutes an essential security interest and, on the other hand, to the assessment of the link between the measure and the conditions set out in the provision.^91^ Therefore, the obligation of good faith requires that WTO members should not use the exceptions in Article XXI of the GATT as a means of circumventing their obligations.^92^ In addition, “the Member invoking the Exception should articulate the essential security interest said […] sufficiently enough to demonstrate their veracity”.^93^

As the Panel’s analysis shows, the self-judging security exception is assessed according to the principle of good faith. This implies that the right of a state to invoke the exception can only be admitted in exceptional circumstances. The state must ensure there is a link between the measure taken and the conditions set out in the exception. The principle of good faith plays an important role in limiting the manner in which the state may invoke the national security interests exception. As in WTO law, so in investment law, good faith could pave the way for an arbitral tribunal to assess the manner in which a self-judging essential security interests provision is implemented by a state.

## Conclusion

The foreign direct investment screening mechanism is a national instrument that a state may create to protect what it considers to be a national security interest. This mechanism takes the form of state measures targeting foreign investment relating to certain categories of the national economy by restricting or prohibiting FDI from entering its territory. This mechanism has evolved over the last decade. In the context of the COVID-19 pandemic, the trend became even more pronounced. The right of states to take measures to safeguard their national interests is, however, limited at the international level by IIAs and customary international law because of its possible impact on investors’ rights. A narrow drafting of the national interest exception and restrictive review in the light of the principle of good faith could ensure both respect for the exercise of state sovereignty and investor protection and thus pave the way for a better balance between the rights of states and investors.

## Footnotes

1 OECD and UNCTAD, Twenty-first Report on G20 Investment Measures, 24 June 2019 (“this development is set to continue in the near future, given firm announcements or plans for further changes in this area in a number of G20 economies”, p. 2).

2 For the differences between SOEs and SWFs, see Bassan (2011), pp. 21–23.

3 Gilson and Milhaupt Curtis (2009), pp. 345–362; Bassan (2011), pp. 21–23; Sornarajah (2011), pp. 267–288.

4 This is the case of the Comprehensive Economic and Trade Agreement (CETA) for instance.

5 For a list of issues or threats covered in selected national security plans, see OECD Study on Security-Related Terms in International Investment Law and in National Security Strategies, OECD 2009, p. 14, Table 2.

6 Shima (2015), 10.1787/5js7svp0jkns-en.

7 OECD, Investment Policy related to National Security. Notification by Lithuania, 23 October 2018, DAF/INV/RD (2018), p. 6. https://www.oecd.org/officialdocuments/publicdisplaydocumentpdf/?cote=DAF/INV/RD(2018)6&docLanguage=En.

8 Australia, “Government Statement: Mining Interests in the Woomera Prohibited Area”, Minister of Defence press release, 17 May 2010. “On 17 May 2010, the Minister of Defence (Australia) announced that any prospective mining investment proposals in the Woomera Prohibited Area, a weapon testing range, where foreign involvement is a factor, and requires Foreign Investment Review Board (FIRB) approval, applicants should first seek assessment from the Defence Department before making any application to the FIRB”, OECD and UNCTAD, Third Report on G20 Investment Measures, 14 June 2010, Annex 1.

9 Among many examples, let us mention that of the Kuwait Investment Authority (1982), the Korea Investment Corporation (2005) and the Norwegian Government Pension Fund (2005).

10 OECD (2018), Ownership and Governance of State-Owned Enterprises: A Compendium of National Practice; OECD, Corporate Governance of SOEs: Guidance and Research, 2011–2017; OECD Corporate Governance Series; OECD (2016), State-Owned Enterprises as Global Competitor: A Challenge or an Opportunity; OECD (2015), OECD Guidelines on Corporate Governance of State-Owned Enterprises.

11 OECD (2016), Risk Management by State-Owned Enterprises and their Ownership, October 2016.

12 France, Décret n° 2019 – 1590 du 31 décembre 2019 relatif aux investissements étrangers en France, JORF, 2 January 2010.

13 S. Eddi Stones & Mason Hayes, China’s Acquisition of Piraeus Port in Greece, as in 2016 COSCO Group & China Shipping Group merged to create China COSCO Shipping, https://is.muni.cz/el/1421/podzim2018/KSCB163/piraeusportpresentation.pdf.

14 Barboza, Chinese Company Ends Unocal Bid, Citing Political Hurdles, New York Times 2 August 2005. More examples are given by Carney (2018), p. 14.

15 OECD and UNCTAD, Twenty-first Report on G20 Investment Measures, 24 June 2019. http://www.oecd.org/investment/investment-policy/21st-Report-on-G20-Investment-Measures.pdf.

16 UNCTAD Report on National Security-Related Screening Mechanisms for Foreign Investment, December 2019, Table 1, pp. 9 and 10. For a survey of country practices related to national security, see Wehrlé and Pohl (2016); For an analysis of EU framework for foreign direct investment control, see Bourgeois (2019); For a detailed analysis of the EU FDI regulatory regime, in particular the German scheme as well as concrete examples of the potential implementation of the screening mechanisms, see Garrod et al. (2020).

17 France, Décret n°2012-691 du 7 mai 2012 relatif aux investissements étrangers soumis à autorisation préalable, Decree n°2012-691 du 7 mai 2012 relatif aux investissements étrangers soumis à autorisation préalable. OECD, UNCTAD and WTO, Eight Report on G20 Trade and Investment Measures (mid-May to mid-October 2012), 31 October 2012, http://www.oecd.org/daf/inv/8thG20report.pdf, p. 51.

18 France, Décret n° 2018-1057 du 29 novembre 2018 relatif aux investissements étrangers soumis à autorisation préalable, JORF, 1 December 2018; OECD and UNCTAD, Twenty-first Report on G20 Investment Measures, 24 June 2019.

19 France, Décret n° 2019 – 1590 du 31 décembre 2019 relatif aux investissements étrangers en France, JORF, 2 January 2010.

20 Germany, “Investment policy related to national security”, Notification by Germany to the OECD, 13 February 2019; Neunte Verordnung zur Änderung der Außenwirtschaftsverordnung, 14 July 2017, OECD and UNCTAD, Twenty-first Report on G20 Investment Measures, 24 June 2019.

21 Italy, Law of 11 May 2012, n. 56, Gazzetta Ufficiale della Repubblica italiana No. 111, 14 May 2012, OECD and UNCTAD, Eighth Report on G20 Investment Measures, 31 October 2012.

22 Italy, Law Decree No. 22/2019 of 25 March 2019, passed into law on 13 May 2019, OECD and UNCTAD, Twenty-first Report on G20 Investment Measures, 24 June 2019.

23 Russian Federation, Federal Law No. 155-FZ of 1 July 2017 on amendments to Article 5 of the Federal Law on Privatization of State and Municipal Property and to the Federal Law on Procedures for Foreign Investment in Business Entities of Strategic Importance for National Defence and State Security, “Amendments to law on the privatisation of state property and on procedures for foreign investment in business entities of strategic importance for national defence and state security”, Presidential Executive Office, 1 July 2017. See also “Amendments to laws on foreign investment and procedure for investing in business entities of strategic importance for national defence”, Presidential Executive Office, 19 July 2017. OECD and UNCTAD, Eighteen Report on G20 Investment Measures, 9 November 2017.

24 Canada, “Guidelines on the National Security Review of Investments” and “Attracting global investments to develop world-class companies”, Government of Canada news release, 19 December 2016. OECD and UNCTAD, Seventeenth Report on G20 Investment Measures, 30 June 2017.

25 Bath (2012), p. 34.

26 Australia, “Mining Interests in the Woomera Prohibited Area Government Statement”, Minister of Defence press release, 17 May 2010; OECD and UNCTAD, Third Report on G20 Investment Measures, 14 June 2010, Annex 1.

27 Australia, “New conditions on the sale of Australian electricity assets to foreign investors”, joint media release, by the Treasurer, the Minister for Home Affairs and The Minister for Immigration and Border Protection, 1 February 2018. OECD and UNCTAD, Nineteenth Report on G20 Investment Measures, 4 July 2018.

28 South Africa, Competition Amendment Act, 2018; OECD and UNCTAD, Twenty-first Report on G20 Investment Measures, 24 June 2019.

29 European Union Foreign Investment Screening regulation enters into force, Brussels, 10 April 2019, http://trade.ec.europa.eu/doclib/press/index.cfm?id=2008; European Commission (2018), Screening of Foreign Direct Investment – An EU Framework, https://trade.ec.europa.eu/doclib/docs/2019/february/tradoc_157683.pdf. For a critical approach, Bismuth (2018), pp. 45–60. For an analysis of the European Union Policy on foreign investment prior to this Regulation, see Chaisse J (2012)

30 European Union, Regulation (EU) 2019/452 of the European Parliament and of the Council of 19 March 2019 establishing a framework for the screening of foreign direct investments into the Union, Official Journal of the European Union, 21 March 2019. See also European Parliament, Framework for screening of foreign direct investments into the European Union, OJ C 262, 25.7.2018, p. 94; European Parliament legislative resolution of 14 February 2019 on the proposal for a regulation of the European Parliament and of the Council establishing a framework for screening of foreign direct investments into the European Union (COM(2017)0487 – C8-0309/2017 – 2017/0224(COD)) OJ C 247, 13.7.2018, p. 28, OECD and UNCTAD, Twenty-first Report on G20 Investment Measures, 24 June 2019. See also Garrod et al. (2020), pp. 345–362.

31 Larson et al. (2012), pp. 3–23.

32 Zimmerman (2019), pp. 1267–1303.

33 See https://home.treasury.gov/policy-issues/international/the-committee-on-foreign-investment-in-the-united-states-cfius.

34 Provisions Pertaining to Certain Investments in the United States by Foreign Persons (31 C.F.R. part 800), https://home.treasury.gov/system/files/206/Part-800-Final-Rule-Jan-17-2020.pdf.

35 Provisions Pertaining to Certain Transactions by Foreign Persons Involving Real Estate in the United States (31 C.F.R. part 802), https://home.treasury.gov/system/files/206/Part-802-Final-Rule-Jan-17-2020.pdf.

36 Lowery (2015), p. 413.

37 UNCTAD World Investment Report (2018), Investment and new Industrial Policies, esp. pp. 177–179; UNCTAD, Investment Policy Monitor (IPM) Special Issue on National Security-Related Screening Mechanisms for Foreign Investment, December 2019, p. 11.

38 UNCTAD Investment Policy Monitor (IPM) Special Issue on National Security-Related Screening Mechanisms for Foreign Investment, December 2019, p. 11.

39 France, Décret n° 2019 – 1590 du 31 décembre 2019 relatif aux investissements étrangers en France, JORF, 2 January 2010.

40 This is the case in Australia, Italy and Spain, OECD Investment Policy Response to COVID-19, OECD Tackling Coronavirus (COVID-19) Contributing to a Global Effort, 17 April 2020, https://read.oecd-ilibrary.org/view/?ref=129_129922-gkr56na1v7&title=OECD-Investment-Policy-Responses-to-COVID-19, pp. 4–5.

41 European Commission Communication, Coordinated economic response to the COVID-19 Outbreak, COM (2020) 112 final (13 March 2020) https://ec.europa.eu/info/sites/info/files/communication-coordinated-economic-response-covid19-march-2020_en.pdf.

42 European Commission, Communication, Guidance to the Member States concerning foreign direct investment and free movement of capital from third countries, and the protection of Europe’s strategic assets, ahead of the application of Regulation (EU) 2019/452 (FDI Screening Regulation) – 25/3/2020 C (2020) 1981 final https://trade.ec.europa.eu/doclib/docs/2020/march/tradoc_158676.pdf.

43 European Commission, Communication, Coordinated economic response to the COVID-19 Outbreak, COM (2020) 112 final (13 March 2020) https://ec.europa.eu/info/sites/info/files/communication-coordinated-economic-response-covid19-march-2020_en.pdf, emphasis in original.

44 European Commission, Communication, Coordinated economic response to the COVID-19 Outbreak, COM (2020) 112 final (13 March 2020), https://ec.europa.eu/info/sites/info/files/communication-coordinated-economic-response-covid19-march-2020_en.pdf.

45 Article 65(1)(b) of the TFEU provides: “The provisions of Article 63 shall be without prejudice to the right of Member States […] to take all requisite measures to prevent infringements of national law and regulations […] or *to take measures which are justified on grounds of public policy or public security*”, emphasis added.

46 Sweden and Denmark are among the countries that do not have an FDI screening mechanism.

47 European Commission, Communication, Guidance to the Member States concerning foreign direct investment and free movement of capital from third countries, and the protection of Europe’s strategic assets, ahead of the application of Regulation (EU) 2019/452 (FDI Screening Regulation) – 25/3/2020 C (2020) 1981 final https://trade.ec.europa.eu/doclib/docs/2020/march/tradoc_158676.pdf.

48 As stated in the Annex to the Guidance, p. 2. European Commission, Communication, Guidance to the Member States concerning foreign direct investment and free movement of capital from third countries, and the protection of Europe’s strategic assets, ahead of the application of Regulation (EU) 2019/452 (FDI Screening Regulation) – 25/3/2020 C (2020) 1981 final https://trade.ec.europa.eu/doclib/docs/2020/march/tradoc_158676.pdf.

49 Press Note 3 (2010 series) dated 17 April 2020 “Press Note: Review of Foreign Direct Investment (FDI) policy for curbing opportunistic takeovers/acquisitions of Indian companies due to the current COVID-19 pandemic”. It amended paragraph 3.1.1 of the 2017 FDI Policy. It establishes restrictions on entities from a country that shares a land border with India, in addition to restrictions imposed on citizens of Pakistan or entities incorporated in Pakistan. Restrictions also apply to the “beneficial owner” of an investment in India who is, however, located in or is a citizen of one of the countries affected by the new measures. Paragraph 3.1.1(a) in its revised version states: “A non-resident entity can invest in India, subject to the FDI Policy except in those sectors/activities which are prohibited. However, an entity of a country, which shares land border with India or where the beneficial owner of an investment into India is situated in or is a citizen of any such country, can invest only under the Government route. Further, a citizen of Pakistan or an entity incorporated in Pakistan can invest, only under the Government route, in sectors/activities other than defence, space, atomic energy and sectors/activities prohibited for foreign investment”.

50 For a very interesting analysis and illustration, see OECD, Study on Security-Related Terms in International Investment Law and in National Security Strategies, 2009. See also Yannaca-Small (2007), p. 98 and Titi (2014), p. 76.

51 According to Article 2(3) of the Hungary-Russia BIT (1995), the “Agreement shall not preclude the application of either Contracting Party measures, necessary for the maintenance of defence, national security and public order, protection of the environment, morality and public health”.

52 According to Article 6.12(1) of the Indian-Singapore Economic Cooperation Agreement (2005), “[n]othing in this Chapter shall be construed […] to require a Party to furnish any information, the disclosure of which it considers contrary to its essential security interests; or to prevent a Party from taking any action which it considers necessary for the protection of its essential security interests”.

53 For the highly complex and varied use of the term “public order”, see OECD, Security-Related Terms in International Investment Law and in National Security Strategies, 2009, pp. 8–10.

54 The recommendation was adopted by the OECD Council on July 16, 1986 at its 646th meeting.

55 Article 3 of the German Model Treaty (2009) on “National and most-favoured nation treatment” states: (a) The following shall more particularly, though not exclusively, be deemed “activity” within the meaning of Article 3 (2): the management, maintenance, use, enjoyment and disposal of an investment. The following shall, in particular, be deemed “treatment less favourable” within the meaning of Article 3: unequal treatment in the case of restrictions on the purchase of raw or auxiliary materials, of energy or fuel or of means of production or operation of any kind, unequal treatment in the case of impeding the marketing of products inside or outside the country, as well as any other measures having similar effects. Measures that have to be taken for reasons of public security and order, public health or morality shall not be deemed “treatment less favourable” within the meaning of Article 3.

56 Article IX(1) of the Estonia-United States BIT. The same approach was adopted, for example, in Article IX of the Latvia-USA BIT; Article IX of the Poland-USA BIT, Article XII (Reservations of Rights) of the Romania-Egypt BIT; Article 2 of the Russia-Hungary BIT. See Yannaca-Small (2007), p. 116.

57 On essential security interests exceptions, see Titi (2014), pp. 76 et seq. See also Yannaca-Small (2007), p. 98.

58 *CMS Gas Transmission Co. v. Argentina*, ICSID Case No. ARB/01/8, Award, 12 May 2005, para. 370. *Contra*: Burke-White and von Staden (2008), pp. 381 et seq.

59 *CMS Gas Transmission Co. v. Argentina*, ICSID Case No. ARB/01/8, Award, 12 May 2005, para. 366 (“the State adopting the measures in question is the sole arbiter of the scope and application of that rule, or whether the invocation of necessity, emergency or other essential security interests is subject to some form of judicial review”).

60 As this issue is not developed further in this chapter, we invite the reader to refer for further analysis to Titi (2014), p. 190 et seq.; Schill and Briese (2009), p. 69; Muchlinski (2009), pp. 59 et seq.

61 Titi (2014), p. 196; Schill and Briese (2009), p. 109.

62 *LG & E Energy Corp., LG & E Capital Corp., and LG & E International, Inc. v. Argentina*, ICSID Case No. ARB/02/1, Decision on Liability, 3 October 2006, para. 214 (“Were the Tribunal to conclude that the provision is self-judging, Argentina’s determination would be subject to a good faith review anyway”); *Continental Casualty Company v. Argentina*, ICSID Case No. ARB/03/9, Award, 5 September 2008, para. 182 (“If Article XI [of the US-Argentina BIT] granted unfettered discretion to a party to invoke it,” this discretion would be subject to “good faith,” while preventing a Tribunal “from entering further into the merits”); *Enron Corporation and Ponderosa Assets, LP v. Argentina*, ICSID Case No. ARB/01/3, Award, 22 May 2007, para. 339 (stating “that Article XI is not self-judging and that judicial review in its respect is not limited to an examination of whether its invocation, or the measures adopted, were taken in good faith”).

63 The same approach is that of Article 2102 of NAFTA on “National Security”, which provides: “1. Subject to Articles 607 (Energy – National Security Measures) and 1018 (Government Procurement Exceptions), nothing in this Agreement shall be construed: […] (b) to prevent any Party from taking any actions that it considers necessary for the protection of its essential security interests (i) relating to the traffic in arms, ammunition and implements of war and to such traffic and transactions in other goods, materials, services and technology undertaken directly or indirectly for the purpose of supplying a military or other security establishment, (ii) taken in time of war or other emergency in international relations, or (iii) relating to the implementation of national policies or international agreements respecting the non-proliferation of nuclear weapons or other nuclear explosive devices”.

64 Article XIV (bis) of the General Agreement on Trade in Services (GATS) states that “[n]othing in this Agreement shall be construed […] to prevent any Member from taking action which it considers necessary for the protection of its essential security interests”.

65 Article 3 of the OECD Code of Liberalisation of Current Invisible Operations (2019).

66 Article 3 of the OECD Codes of Liberalisation of Capital Movements (2019).

67 The OECD Recommendation of the Council on Member Country Measures concerning National Treatment of Foreign-Controlled Enterprises in OECD Member Countries and Based on Considerations of Public Order and Essential Security Interest*,* OECD/LEGAL/0226 http://legalinstruments.oecd.org.

68 The National Treatment’s instrument is a non-binding and voluntary undertaking by adhering and non-adhering OECD countries https://www.oecd.org/daf/inv/investment-policy/nationaltreatmentinstrument.htm.

69 Guidelines for Recipient Country Investment Policies Relating to National Security, Recommendation adopted by the OECD Council on 25 May 2009, and the same text is found in the OECD, Recommendation of the Council on Guidelines for Recipient Country Investment Policies relating to National Security, OECD/LEGAL/0372.

70 OECD, Recommendation of the Council on Guidelines for Recipient Country Investment Policies relating to National Security, OECD/LEGAL/0372.

71 Annex on Guidelines for Recipient Country Investment Policies Relating to National Security, in OECD, Recommendation of the Council on Guidelines for Recipient Country Investment Policies relating to National Security, OECD/LEGAL/0372.

72 As stated in the Annex “*Codification and publication*. Primary and subordinate laws should be codified and made available to the public in a convenient form (e.g. in a public register; on internet). In particular, evaluation criteria used in reviews should be made available to the public”.

73 OECD, Recommendation of the Council on Guidelines for Recipient Country Investment Policies relating to National Security, OECD/LEGAL/0372.

74 OECD, Recommendation of the Council on Guidelines for Recipient Country Investment Policies relating to National Security, OECD/LEGAL/0372.

75 As stated in the Annex on Guidelines for Recipient Country Investment Policies relating to National Security, in OECD, Recommendation of the Council on Guidelines for Recipient Country Investment Policies relating to National Security, OECD/LEGAL/0372: “All countries share a collective interest in maintaining international investment policies that are open, legitimate and fair. Through various international standards, governments recognise this collective interest and agree to participate in related international accountability mechanisms (e.g. the OECD notification and peer review obligations in relation to restrictive investment policies). In particular, these help constrain domestic political pressures for restrictive and discriminatory policies. Recipient governments should participate in and support these mechanisms”.

76 OECD, Note on Current and Emerging Trends on “Acquisition – and Ownership – Related Policies to Safeguard Essential Security Interests. New Policies to Manage New Threats”, 12 March 2019.

77 UNCTAD (2009), p. 72.

78 Article 18(4)(b) of the Canadian Model BIT (2014).

79 See also Article 4 of the Belgian-Luxembourg Economic Union (BLEU)-China BIT (2005).

80 Article 4 of the China-Philippines BIT (1992).

81 WTO Panel Report, *Russia – Measures concerning traffic in transit*, 5 April 2019. The Panel was composed of Georges Abi-Saab (Chairperson), Ichiro Araki and Mohammad Saeed (members). As the Panel was composed of eminent members, its reasoning makes it a reference in this matter.

82 WTO Panel Report, *Russia – Measures concerning traffic in transit*, 5 April 2019, para. 7.5.3.1.

83 WTO Panel Report, *Russia – Measures concerning traffic in transit*, 5 April 2019, para. 7.5.4.

84 WTO Panel Report, *Russia – Measures concerning traffic in transit*, 5 April 2019, para. 7.5.3.1.1.

85 WTO Panel Report, *Russia – Measures concerning traffic in transit*, 5 April 2019, para. 7.5.6.

86 Article XXI of the GATT provides: Nothing in this Agreement shall be construed

- to require any contracting party to furnish any information the disclosure of which it considers contrary to its essential security interests; or
- to prevent any contracting party from taking any action which it considers necessary for the protection of its essential security interests
  (i) relating to fissionable materials or the materials from which they are derived;
  (ii) relating to traffic in arms, ammunition and implement of war and to such traffic in other goods and materials as is carried on directly or indirectly for the purpose of supplying a military establishment;
  (iii) taken in time of war or other emergency in international relations; or
- to prevent any contracting party from taking any action in pursuance of its obligations under the United Nations Charter for the maintenance of international peace and security.

87 Article 32(1) of the Vienna Convention on the Law of Treaties states that “[a] treaty shall be interpreted in good faith in accordance with the ordinary meaning to be given to the terms of the treaty in their context and in the light of its object and purpose”.

88 WTO Panel Report, *Russia – Measures concerning traffic in transit*, 5 April 2019, para. 7.130, p. 56.

89 WTO Panel Report, *Russia – Measures concerning traffic in transit*, 5 April 2019, para. 7.131, p. 56.

90 WTO Panel Report, *Russia – Measures concerning traffic in transit*, 5 April 2019, para. 7.132.

91 WTO Panel Report, *Russia – Measures concerning traffic in transit*, 5 April 2019, para. 7.138.

92 WTO Panel Report, *Russia – Measures concerning traffic in transit*, 5 April 2019, para. 7.133.

93 WTO Panel Report, *Russia – Measures concerning traffic in transit*, 5 April 2019, para. 7.134.

## References

[CR1] Bassan F (2011) The law of sovereign wealth funds. Edward Elgar

[CR2] Bath V (2012). Foreign investment, the national interest and national security - foreign direct investment in Australia and China. Sydn Law School Rev.

[CR3] Bismuth R (2018) Screening the Commission’s regulation proposal establishing a framework for screening FDI into the EU. Eur Invest Law Arbitr Rev 2018:45–61

[CR4] Bourgeois JHJ (ed) (2019) EU framework for foreign direct investment control, European Monographs. Wolters Kluwer

[CR5] Burke-White WW, von Staden A (2008) Investment protection in extraordinary times: the interpretation and application of non-precluded measures provisions in bilateral investment treaties. Va J Int Law 48:307–410

[CR6] Carney R (2018) Authoritarian capitalism. Sovereign wealth funds and state-owned enterprises in East Asia and beyond. Cambridge University Press

[CR7] Chaisse J (2012) Promises and pitfalls of the European Union policy on foreign investment – how will the new EU competence on FDI affect the emerging global regime? J Int Econ Law 68

[CR8] Garrod D, Casselbrant-Multala S, Garritsen L (2020) Foreign investment: an overview of EU and national case law, e-competition special issue Foreign Investment, 16 January

[CR9] Gilson R, Milhaupt Curtis J (2009) Sovereign wealth funds and corporate governance: a minimalist response to the new mercantilism. Revue d’économie financière (English ed.). Hors-série, 2009. Sovereign wealth funds: Special Issue 2009:345–362. https://www.persee.fr/doc/ecofi_1767-4603_2009_hos_9_1_5520

[CR10] Larson A, Fagan D, Berengaut A, Plotkin M (2012) Lessons from CFIUS for national security reviews of foreign investment. In: Sauvant K, Sachs L, Schmit Jonbloed W (eds) Sovereign investment: concerns and policy reactions. Oxford University Press

[CR11] Lowery C (2015) The US approach to sovereign wealth funds and the role of CFIUS. In: Sauvant K, Sachs L, Schmit Jongbloed W (eds) Sovereign investment: concerns and policy reactions. Oxford University Press

[CR12] Muchlinski P (2009) Trends in international investment agreements: balancing investor rights and the right to regulate – the issue of national security. In: Sauvant KP (ed) Yearbook on International Investment Law & Policy 2008–2009. Oxford University Press, New York

[CR14] Schill S, Briese R (2009). “If the state considers”: self-judging clauses in international dispute settlement. Max Planck UNYB.

[CR15] Shima Y (2015) The policy landscape for international investment by government-controlled investors: a fact-finding survey, OECD Working Papers on International Investment, 2015/01. OECD Publishing

[CR16] Sornarajah M (2011). Sovereign wealth funds and the existing structure of the regulation of investments. Asian J Int Law.

[CR17] Titi C (2014) The right to regulate in international investment law. Nomos and Hart Publishing

[CR18] UNCTAD (2009) The protection of national security in IIAs

[CR19] Wehrlé F, Pohl J (2016) Investment policies related to national security: a survey of country practices, OECD Working Papers on International Investment, n° 2016/02. OECD Publishing, Paris

[CR20] Yannaca-Small K (2007) Part. I, Chapter 5: essential security interests under international investment law in international investment perspectives: freedom of investment in a changing world. OECD Publishing

[CR21] Zimmerman E (2019). The foreign investment risk review modernization act: how CFIUS became a tech office. Berkeley Technol Law J.

